# Prognostic value of isolated tumor cells and micrometastasis of lymph nodes in invasive urinary bladder cancer

**DOI:** 10.1371/journal.pone.0302445

**Published:** 2024-10-25

**Authors:** Harin Cheong, Youngeun Yoo, Sun Hee Sung, Sanghui Park, Dong Hyeon Lee, Kyoung Ae Kong, Heae Surng Park, Min-Sun Cho

**Affiliations:** 1 Department of Forensic Medicine, College of Medicine, The Catholic University of Korea, Seoul, Republic of Korea; 2 Department of Pathology, Ewha Womans University Seoul Hospital, Seoul, Republic of Korea; 3 Department of Pathology, Ewha Womans University Mokdong Hospital, Seoul, Republic of Korea; 4 Department of Urology, Ewha Womans University Mokdong Hospital, Seoul, Republic of Korea; 5 Department of Preventive Medicine, Ewha Womans University College of Medicine, Seoul, Republic of Korea; University of Pisa, ITALY

## Abstract

**Introduction:**

The prognostic significance of nodal micrometastasis and isolated tumor cells (ITC) in urinary bladder cancer (UBC) is unknown. We aimed to evaluate the prevalence, clinical impact, and clinicopathological characteristics of nodal micrometastasis and ITC in UBC.

**Materials and methods:**

A total of 124 patients with UBC undergoing surgery were investigated. Detection of micrometastasis and ITC was performed using pancytokeratin immunohistochemistry (IHC). Histopathologic and clinical findings were correlated with patients’ outcome.

**Result:**

IHC detected nodal micrometastasis and ITC (pNmi group) in 12.9% (13/101) of originally node-negative patients and in 26.1% (6/23) of originally node-positive patients (pN+ group). The remaining 88 were truly node-negative patients (pN0 group). After IHC, all 13 patients in the pNmi group were upstaged from pN0 to pN1-2 and one patient in the pN+ group was changed from pN1 to pN2. Nodal micrometastasis and ITC were significantly associated with mixed urothelial carcinoma (UC) (*p* = 0.002), UC with discohesive pattern (*p* = 0.006), glandular differentiation (*p* = 0.043), lymphovascular invasion (*p* = 0.009), and budding-like tumor cell clusters (*p* = 0.002). The pNmi group had significantly worse cancer-specific survival than the pN0 group in univariate (*p* = 0.004) and multivariate (*p* = 0.040) analysis.

**Conclusion:**

IHC frequently identified nodal micrometastasis and ITC in originally node-negative UBC patients on routine pathological examination. Nodal micrometastasis and ITC were independently associated with cancer-related mortality in UBC. IHC might be selectively used to detect micrometastasis and ITC in UBC having specific pathological features.

## Introduction

Determining lymph node (LN) metastasis in surgically resected specimen is an important part of pathological examination. It was usually determined by examination on hematoxylin and eosin (H&E)-stained slides. Due to easy accessibility of immunohistochemistry (IHC), immunostaining for cytokeratin can lead to discovery of very small-sized nodal metastases, such as isolated tumor cells (ITC) and micrometastasis. ITC is defined as a single cell or a cluster of tumor cells with fewer than 200 cells or less than 0.2 mm in diameter with little stromal reaction: micrometastasis is a metastasis with a size that is greater than 0.2 mm but less than 2 mm, according to the 8th edition of the American Joint Committee on Cancer (AJCC) staging system [1]. This definition of ITC and micrometastasis is generally applied to cancers of all organs, but nodal staging differs by cancer site. ITCs are staged as N1 or higher in melanoma and Merkel cell carcinoma, whereas in breast and gynecological cancers, ITCs are staged as N0(i+) [1]. Micrometastasis was first applied to the breast cancer staging system and reported as pN1mi. However, micrometastasis to the other organ has been considered pN1 so far. The significance of ITC and micrometastasis in many cancer sites are unknown and further studies are needed.

Urinary bladder cancer (UBC) is the 10^th^ most common and 13^th^ most deadly cancer in the world [2]. Standard treatment for muscle-invasive UBC includes radical cystectomy (RC) and lymphadenectomy along with or without neoadjuvant chemotherapy according to the NCCN guideline. Adjuvant chemotherapy (AC) is performed in UBC patients with extravesical extension or lymph node metastasis [3, 4]. There is no established guideline for ITC or micrometastasis in UBC. Only a few studies have been presented on ITC and micrometastasis in UBC detected using IHC with reported prevalence of 3.3–13.7% [5–8]. Results of these studies are limited because of a small number of study cohort, a short follow-up period, and an insufficient prognostic impact.

In this study, nodal micrometastasis and ITC were investigated using IHC in a relatively large-sized UBC cohort who underwent RC with lymphadenectomy. We evaluated influence of micrometastasis and ITC on TNM staging and clinical outcome with related clinicopathological characteristics.

## Materials and methods

### 1. Case selection

Patients who received RC with lymph node dissection (LND) for invasive UBC between January 2013 and December 2015 at Ewha Womans University Mokdong Hospital (Seoul, Republic of Korea) were eligible for this study. We excluded patients who received neoadjuvant chemotherapy or had non-urothelial carcinoma histology (n = 5) or died shortly after operation (n = 2) owing to surgical complication such as post-operative infection and cardiovascular event. We excluded 2 cases of squamous cell carcinoma, 2 cases of adenocarcinoma, and one case of small cell carcinoma in this study, since the pathogenesis and clinical course are different between urothelial and non-urothelial carcinoma. Clinical data including age, sex, status of adjuvant chemotherapy (AC), data related to recurrence and metastasis, follow-up data, and survival outcome were assessed by reviewing electronic medical records.

A total of 124 cases of invasive UBC treated with radical surgery were included in this study. The operation was done by three urologic surgeons with similar cystectomy and standard pelvic lymph node dissection method. AC was given within 12 weeks after surgery in patients with histologically confirmed locally advanced disease (pT3 or pT4) or regional LN metastases (pN+) or suspected residual tumor (incomplete surgery) or presence of lymphovascular invasion. The AC protocol included three cycles of gemcitabine and cisplatin for pT3/4 disease and six cycles of the same regimen for pN+ disease. For patients with metastasis or advanced condition during the follow up period, other regimens including methotrexate, vinblastine, adriamycin, and/or cisplatin were applied. The patients were followed by every 3 to 4 months for the first two years and every 6 to 12 months until 5 years.

Pathological data were evaluated by reviewing all glass slides of transurethral resection (TUR) and RC specimens. Light microscopic examination was performed using the original H&E slide. The pathologic tumor (pT), node, (pN) and TNM stage were determined based on the 8^th^ edition of AJCC staging system. This study was approved by the Institutional Review Board (IRB) of Ewha Womans University Mokdong Hospital (protocol no. 2018-8-049). The requirement for informed consent was waived by the IRB due to its retrospective nature. Clinical data were collected from electronic medical records between December 18, 2018 and December 12, 2021, and were completely anonymized.

### 2. Immunohistochemistry

Pancytokeratin IHC was performed for all LNs with negative results on initial pathological diagnosis. Only one additional section was cut for IHC. IHC for pan-cytokeratin (1:100, monoclonal, Novocastra, Newcastle, UK) was performed using a BOND-MAX autoimmunostaining system (Leica Biosystem, Melbourne, Australia) with BOND^TM^ Polymer Refine Detection Kit DS9800 (Leica Biosystem, Melbourne, Australia), as CK IHC has been reported to be a sensitive method for detecting micrometastasis or ITC in axillary node-negative breast cancer [9]. Sections (4-μm-thick) from formalin-fixed, paraffin embedded pretreatment tumor biopsy specimens were transferred to adhesive slides and dried at 62°C for 30 min. Slides were then deparaffinized. Endogenous peroxidase was quenched by incubating the tissues with 0.3% hydrogen peroxide for 10 min. Antigen retrieval was performed using the BOND Epitope Retrieval solution for 20 min at 97°C. Sections were incubated with primary antibodies for 15 minutes, the post-primary antibody for 10 min, and the polymer for 30 min, followed by expression with 3,3’-diaminobenzidine and counterstaining with hematoxylin.

### 3. Pathological evaluation of micrometastasis and ITC and grouping of pN stage

**Fig 1:**
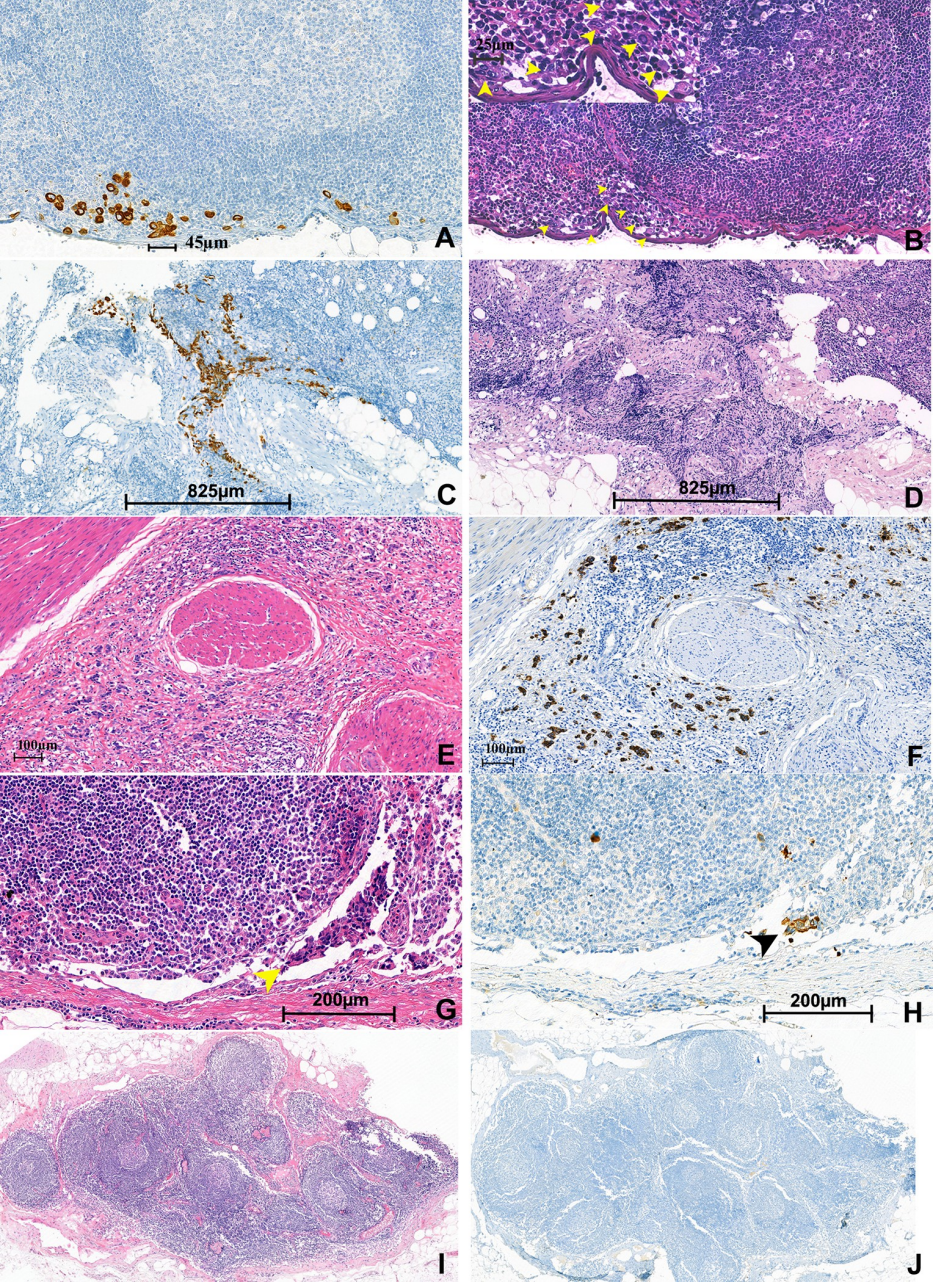
Detection of micrometastasis and isolated tumor cells (ITC) in lymph nodes of urinary bladder cancer patients. A case (A, B) of urothelial carcinoma with nodal ITC. ITCs were highlighted on pan-cytokeratin immunohistochemistry. The largest cell cluster measured 45μm (A). The original hematoxylin and eosin (H&E) slide showed a few atypical cells resembling histiocytes corresponding to ITCs (arrowheads; inlet, magnification of ITCs, 400x) (B). A case of conventional urothelial carcinoma with nodal micrometastasis (C, D). Micrometastasis was clearly visible on the immunostained slide (C). The original H&E slide had a squeezing artifact and ITCs were difficult to discriminate (D). A case of mixed urothelial carcinoma with discohesive pattern and budding-like clusters that had ITCs (E, F, G, H). Discohesive tumor cells infiltrated bladder proper muscle (E), which were highlighted by immunostaining (F). ITC (arrowhead) was noted in the right iliac lymph node on the original H&E (G), which was reduced in the deeper section of the immunohistochemical stain (H). A negative control case with H&E (I) and immunostained slides (J). (A, B, G, and H, 200x magnification; C, D, E, and F, 100x magnification; H and I, 50x magnification).

Nodal micrometastasis and ITC were detected by IHC and determined with the following criteria in the breast cancer section of the 8^th^ edition of AJCC cancer staging system [1]. ITC was defined as presence of a single tumor cell or malignant cell clusters which is no larger than 0.2 mm in diameter. Micrometastasis was defined as the presence of tumor cells which is larger than 0.2 mm but not larger than 2.0 mm in diameter. In this study, presence of ITC or micrometastasis in a LN was considered together as “occult LN metastasis” (Fig 1). Most lymph nodes were longitudinally sectioned and stained with H&E.

Immunostained slides were evaluated by two pathologists (HC and MC) without knowledge of clinicopathological information. A few discordant IHC results were discussed and reviewed with two pathologists and a consensus diagnosis was reached. To evaluate prognostic impact of occult LN metastasis, the study population was grouped based on the new pN stage after IHC: pN0 (patients with no metastasis after IHC), pNmi (patients with occult LN metastasis after IHC who were originally pN0 on the initial diagnosis with routine H-E slide examination), and pN+ (patients with nodal metastasis on both initial and IHC diagnoses) groups.

### 4. Pathological evaluation of histological variants and features

Pathological evaluation of histological variants was performed according to the 2016 WHO classifications of urothelial tract tumor [10]. In the present study, all existing histological subtypes (variants) and divergent differentiation of urothelial carcinoma (UC) were specified, including squamous differentiation, glandular differentiation, micropapillary, plasmacytoid, sarcomatoid, nested, lymphoepithelioma-like, and microcystic variants. The existence of more than one histological variant type in addition to conventional UC was designated as mixed UC. More than two histological variant types in one case was regarded as multiple variant differentiation. The tumor differentiation was graded to low and high grades. The budding-like tumor cell clusters (budding-like clusters) was defined as isolated single tumor cells or small clusters composed of fewer than 20 cells near tumor border in more than one field of 200x magnification (Fig 1D). These clusters were also present in the retraction artifact and were present as small lumps in the interstitial tissue. These areas were roughly estimated. Necrosis was considered significant when the necrotic portion was more than 50% of total tumor area.

### 5. Outcome and statistical analysis

Disease recurrence was defined as local tumor recurrence within urinary tracts and/or regional lymph nodes and/or distant metastasis. Recurrence-free survival (RFS) was defined as the time from RC to recurrence or the last follow-up or death of any cause. Cancer-specific survival (CSS) was defined as the time from RC to the last follow-up or death from UBC. Overall survival (OS) was defined as the time from RC to the last follow-up or death of any cause.

Distributions of categorical variables between the groups were compared by the chi-square or Fisher exact tests. Continuous variables were compared by Student’s *t*-test or Mann-Whitney test. Survival curves were estimated by the Kaplan-Meier method compared by log-rank tests. Univariate and multivariate Cox proportional hazard regression models were used to evaluate the risk of each clinicopathological parameter for disease recurrence, cancer-specific death and overall survival. *P* ≤ 0.05 was considered statistically significant. All statistical tests were performed using IBM SPSS software version 23.0.0 (SPSS Inc., Chicago, IL, USA).

## Results

### 1. Clinicopathologic characteristics of patients

Table 1 summarizes the clinicopathologic characteristics of 124 patients. Their median age was 63.7 years old (range, 27–87 years; standard deviation [SD] ±10.4). Patients were predominantly males (83.2%, 102 out of 124). Histologic type of carcinoma was comprised of pure conventional UC in 54.0% (67 of 124) and mixed UC in 46.0% (57 of 124). Among mixed UC, 7.3% (9 of 124) showed multiple variant histology. Micropapillary variant (n = 14, 11.3%) was the most common histological variant followed by squamous differentiation (n = 13, 10.5%), plasmacytoid variant (n = 7, 5.6%), discohesive pattern (n = 4, 3.2%), glandular differentiation (n = 3, 2.4%) and nested variant (n = 3, 2.4%) among mixed UC with one variant histology. Other rare variants of UC included lymphoepithelioma-like (n = 1), microcystic (n = 1), and sarcomatoid (n = 1) variants in the study cohort. Among histologic features, budding-like cluster was observed in 66.1% (82 of 124). Lymphovascular invasion was observed in 50 (40.3%) patients. Perineural invasion was present in 16 (12.9%) patients (Table 2). AC was performed in 32.3% (40 of 124). No patient received AC among the pT1 group. Six among pT2 (14.3%), 29 among pT3 (82.9%), and 5 patients among pT4 (83.3%) received AC. Seven patients among compatible condition for AC (pT3, 6 patients; pT4, 1 patient) did not take AC. During a median follow-up period of 80 months (range, 3.6–106.5 months; SD ± 27.9 months), 45 patients had tumor recurrence (36.3%), including 24 local recurrence and/or 26 distant metastasis. Overall death was found in 26.6% (33 of 124), and 21.8% (27 of 124) died of UBC.

**Table 1:** Clinicopathologic characteristics of bladder cancer patients with radical cystectomy according to occult LN metastasis.

			All (n = 124)	pN0 group (n = 88)	pNmi group (n = 13)	pN+ group (n = 23)	*p*-values
Age			63.7(±10.4)	63.7(±9.8)	63.5(±14.7)	63.7(±10.6)	
Sex							
	male		102 (82.3%)	75 (85.2%)	10 (76.9%)	17 (73.9%)	0.118
	female		22 (17.7%)	13 (14.8%)	3 (23.1%)	6 (26.1%)	
Histological subtype							
	Pure conventional UC		67 (54%)	55 (62.5%)	2 (15.4%)	10 (43.5%)	0.003
	Mixed UC		57 (46%)	33 (37.5%)	11 (84.6%)	13 (56.5%)	
		Multiple	9 (7.3%)	5 (5.7%)	1 (7.7%)	3 (13%)	
		Micropapillary	14 (11.3%)	7 (8%)	3 (23.1%)	4 (17.4%)	
		Squamous	13 (10.5%)	11 (12.5%)	1 (7.7%)	1 (4.3%)	
		Plasmacytoid	7 (5.6%)	3 (3.4%)	0 (0%)	4 (17.4%)	
		Discohesive	4 (3.2%)	1 (1.1%)	3 (23.1%)	0 (0%)	
		Glandular	3 (2.4%)	1 (1.1%)	2 (15.4%)	0 (0%)	
		Nested	3 (2.4%)	1 (1.1%)	1 (7.7%)	1 (4.3%)	
		Sarcomatous	1 (0.8%)	1 (1.1%)	0 (0%)	0 (0%)	
		Lipogenic	1 (0.8%)	1 (1.1%)	0 (0%)	0 (0%)	
		Lymphoepithelial	1 (0.8%)	1 (1.1%)	0 (0%)	0 (0%)	
		Microcystic	1 (0.8%)	1 (1.1%)	0 (0%)	0 (0%)	
Pathological tumor stage							
	pT1		41 (33.1%)	37 (42%)	4 (30.8%)	0 (0%)	0.000
	pT2		42 (33.9%)	33 (37.5%)	5 (38.5%)	4 (17.4%)	
	pT3		35 (28.2%)	18 (20.5%)	3 (23.1%)	14 (60.9%)	
	pT4		6 (4.8%)	0 (0%)	1 (7.7%)	5 (21.7%)	
Pathological node stage, original (by H&E stain)							
	pN0		101 (81.5%)	101 (100%)	0 (0%)	0 (0%)	0.000
	pN1		11 (8.9%)	0 (0%)	0 (0%)	11 (47.8%)	
	pN2		11 (8.9%)	0 (0%)	0 (0%)	11 (47.8%)	
	pN3		1 (0.8%)	0 (0%)	0 (0%)	1 (4.3%)	
Pathological node stage, new (by IHC)							
	pN0		88 (71%)	88 (100%)	0 (0%)	0 (0%)	0.000
	pN1		20 (16.1%)	0 (0%)	10 (76.9%)	10 (43.5%)	
	pN2		15 (12.1%)	0 (0%)	3 (23.1%)	12 (52.2%)	
	pN3		1 (0.8%)	0 (0%)	0 (0%)	1 (4.3%)	
AJCC TNM stage, original (by H&E stain)							
	I (pT1 pN0)		41 (33.1%)	41 (42%)	0 (0%)	0 (0%)	0.000
	II (pT2 pN0)		38 (30.6%)	38 (37.5%)	0 (0%)	0 (0%)	
	IIIA (pT1-4a pN1)		33 (26.6%)	22 (20.5%)	0 (0%)	11 (47.8%)	
	IIIB (pT1-4a pN2-3)		11 (8.9%)	0 (0%)	0 (0%)	11 (47.8%)	
	IVA (pT4b anyN)		1 (0.8%)	0 (0%)	0 (0%)	1 (4.3%)	
AJCC TNM stage, new (by IHC)							
	I (pT1 pN0)		37 (29.8%)	37 (42%)	0 (0%)	0 (0%)	0.000
	II (pT2 pN0)		33 (26.6%)	33 (37.5%)	0 (0%)	0 (0%)	
	IIIA (pT1-4a pN1)		38 (30.6%)	18 (20.5%)	10 (76.9%)	10 (43.5%)	
	IIIB (pT1-4a pN2-3)		15 (12.1%)	0 (0%)	3 (23.1%)	12 (52.2%)	
	IVA (pT4b anyN)		1 (0.8%)	0 (0%)	0 (0%)	1 (4.3%)	
Locoregional recurrence							
	No		100 (80.6%)	75 (85.2%)	7 (53.8%)	20 (78.3%)	0.027
	Yes		24 (19.4%)	13 (14.8%)	6 (26.2%)	35(21.7%)	
Distant metastasis							
	No		98 (79%)	76 (86.4%)	9 (69.2%)	13 (56.5%)	0.002
	Yes		26 (21%)	12 (13.6%)	4 (30.8%)	10 (43.5%)	
Disease recurrence							
	No		79 (63.7%)	64 (72.7%)	6 (46.2%)	9 (39.1%)	0.004
	Yes		45 (36.3%)	24 (27.3%)	7 (53.8%)	14 (60.9%)	
Cancer-related death							
	No		97 (78.2%)	77 (87.5%)	7 (53.8%)	13 (56.5%)	0.000
	Yes		27 (21.8%)	11 (12.5%)	6 (46.2%)	10 (43.5%)	
Overall death							
	No		91 (73.4%)	71 (80.7%)	7 (53.8%)	13 (56.5%)	0.007
	Yes		33 (26.6%)	17 (19.3%)	6 (46.2%)	10 (43.5%)	
Adjuvant chemotherapy							
	No		84 (67.7%)	74 (84.1%)	10 (76.9%)	0 (0%)	0.000
	Yes		40 (32.3%)	14 (15.9%)	3 (23.1%)	23 (100%)	

UC, urothelial carcinoma; H&E, hematoxylin and eosin; IHC, immunohistochemistry

**Table 2:** Relations of pathologic features with lymph node metastasis including occult LN metastasis.

			All (n = 124)	pN0 group (n = 88)	pNmi group (n = 13)	pN+ group (n = 23)	p-values pN0 vs. pNmi	p-values pN0 vs. pN+
Pure conventional UC			67 (54%)	55 (62.5%)	2 (15.4%)	10 (43.5%)	0.002	0.153
Mixed UC			57 (46%)	33 (37.5%)	11 (84.6%)	13 (56.5%)		
	Multiple variant	Absent	115 (92.7%)	83 (94.3%)	12 (92.3%)	20 (87%)	0.572	0.359
		Present	9 (7.3%)	5 (5.7%)	1 (7.7%)	3 (13%)		
	Micropapillary variant	Absent	110 (88.7%)	81 (92%)	10 (76.9%)	19 (82.6%)	0.118	0.235
		Present	14 (11.3%)	7 (8%)	3 (23.1%)	4 (17.4%)		
	Squamous differentiation	Absent	111 (89.5%)	77 (87.5%)	12 (92.3%)	22 (95.7%)	1.000	0.454
		Present	13 (10.5%)	11 (12.5%)	1 (7.7%)	1 (4.3%)		
	Plasmacytoid variant	Absent	117 (94.4%)	85 (96.6%)	13 (100%)	19 (82.6%)	1.000	0.033
		Present	7 (5.6%)	3 (3.4%)	0 (0%)	4 (17.4%)		
	Discohesive variant	Absent	120 (96.8%)	87 (98.9%)	10 (76.9%)	23 (100%)	0.006	1.000
		Present	4 (3.2%)	1 (1.1%)	3 (23.1%)	0 (0%)		
	Glandular differentiation	Absent	121 (97.6%)	87 (98.9%)	11 (84.6%)	23 (100%)	0.043	1.000
		Present	3 (2.4%)	1 (1.1%)	2 (15.4%)	0 (0%)		
	Nested variant	Absent	121 (97.6%)	87 (98.9%)	12 (92.3%)	22 (95.7%)	0.242	0.373
		Present	3 (2.4%)	1 (1.1%)	1 (7.7%)	1 (4.3%)		
Tumor grade		Low grade	21 (16.9%)	18 (20.5%)	2 (15.4%)	1 (4.3%)	1.000	0.116
		High grade	103 (83.1%)	70 (79.5%)	11 (84.6%)	22 (95.7%)		
Lymphovascular invasion		Absent	74 (59.7%)	67 (76.1%)	5 (38.5%)	2 (8.7%)	0.999	0.000
		Present	50 (40.3%)	21 (23.9%)	8 (61.5%)	21 (91.3%)		
Perineural invasion		Absent	108 (87.1%)	80 (90.9%)	11 (84.6%)	17 (73.9%)	0.613	0.070
		Present	16 (12.9%)	8 (9.1%)	2 (15.4%)	6 (26.1%)		
Necrosis		Absent	100 (80.6%)	73 (83%)	9 (69.2%)	18 (78.3%)	0.260	0.558
		Present	24 (19.4%)	15 (17%)	4 (30.8%)	5 (21.7%)		
Budding-like clusters		Absent	42 (33.9%)	40 (45.5%)	1 (7.7%)	1 (4.3%)	0.013	0.000
		Present	82 (66.1%)	48 (54.5)	12 (92.3)	22 (95.7)		

UC, urothelial carcinoma

### 2. Detection of occult LN metastasis and shift of pN

Standard pathological examination revealed that 23 patients were positive for node metastasis and 101 patients were node negative. IHC newly detected microscopic metastasis in 19 patients. In the originally node-negative group, occult LN metastasis (pNmi) was detected in 12.9% (13 of 101), including 11 ITC and two micrometastasis found in 1 or 2 LNs (mean, 1.2 nodes). The rest of originally node-negative group excluding occult LN metastasis was truly node-negative (pN0, n = 88). In the originally node-positive group (pN+ group, n = 23), 26.1% (6 of 23) were found to have additionally detected LN metastasis comprising 3 ITC and 3 micrometastasis, which were identified in 1 to 4 LNs (average 2.75 nodes).

**Fig 2:**
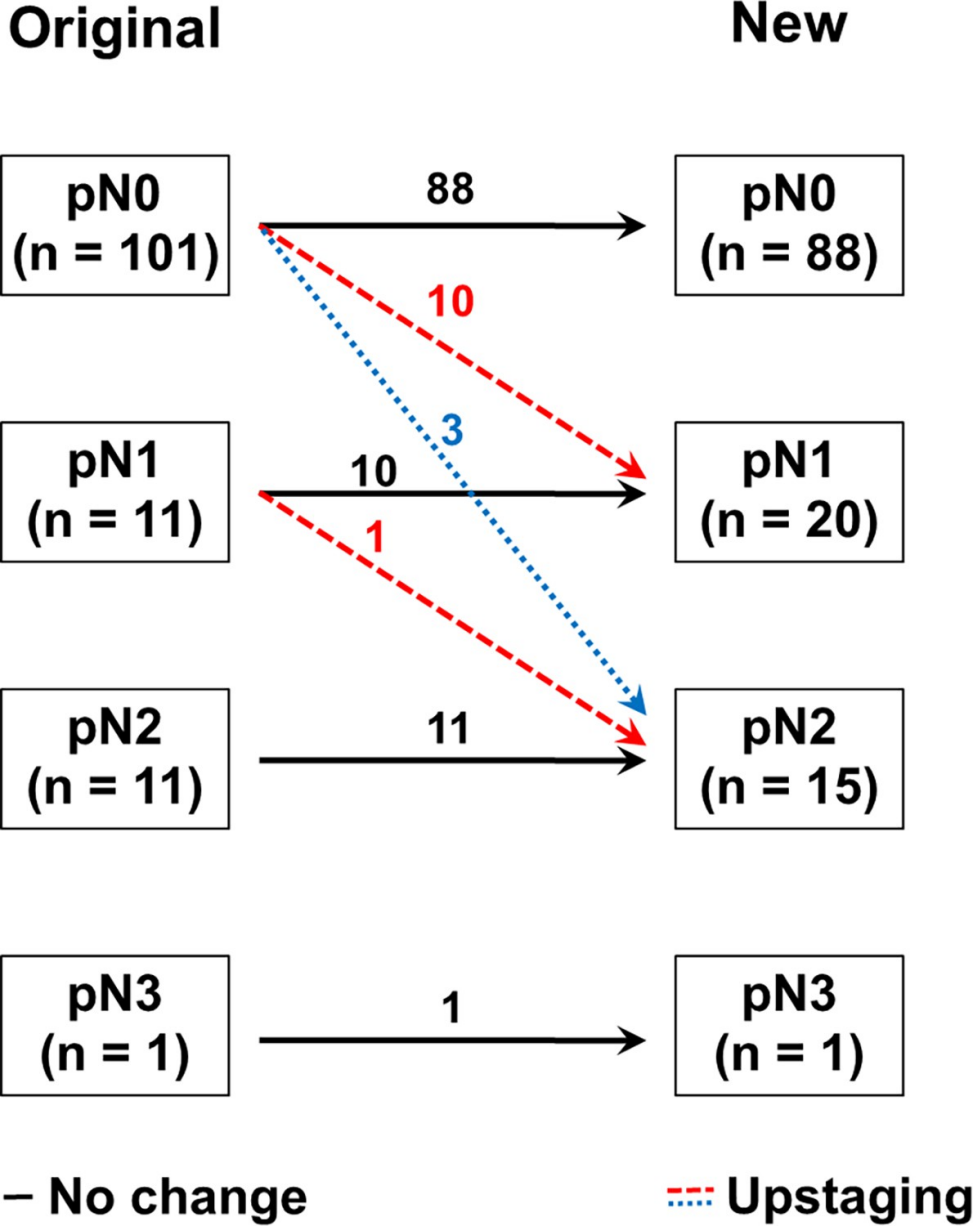
Changes in pN stage after immunohistochemistry.

Changes of pN stage were illustrated in Fig 2. After IHC, pN stage was upstaged in 11.3% (14 of 124) of patients including 10 patients from pN0 to pN1, 3 from pN0 to pN2, and 1 from pN1 to pN2. The average number of resected LN in all patients was 18.6 (range, 1–53; SD ± 9.8). Some specimens labelled “pelvic lymph node” had predominantly fat tissue with few lymph nodes, which could reduce the detection rate of occult metastasis, but there was no significant difference in the average number of resected LNs among pN0, pNmi and pN+ groups (pN0, 17.6; pNmi, 23.2; pN+, 19.7; *p* = 0.134).

### 3. Pathological characteristics of occult LN metastasis

Table 2 summarizes the association of pathologic characteristics and occult LN metastasis. The incidence of occult LN metastasis was significantly higher in mixed UC than in pure conventional UC (*p* = 0.002). Among each histologic variant, discohesive pattern and glandular differentiation were significantly associated with occult LN metastasis (*p* = 0.006 and *p* = 0.043, respectively). Micropapillary variant tended to have occult LN metastasis without reaching statistical significance. Plasmacytoid variant was significantly associated with positive-node metastasis (pN+ vs. pN0; *p* = 0.033), though no case of plasmacytoid variant showed occult LN metastasis. Budding-like clusters showed significant association with occult LN (pNmi vs. pN0; *p* = 0.013) as well as positive-node metastasis (pN+ vs. pN0; *p* < 0.001). Lymphovascular invasion had significant association with positive-node metastasis (pN+ vs. pN0; *p* < 0.001), but not with occult LN metastasis (pNmi vs. pN0; *p* = 0.999).

4. Prognostic significance of occult LN metastasis and other clinicopathologic parameters

**Fig 3:**
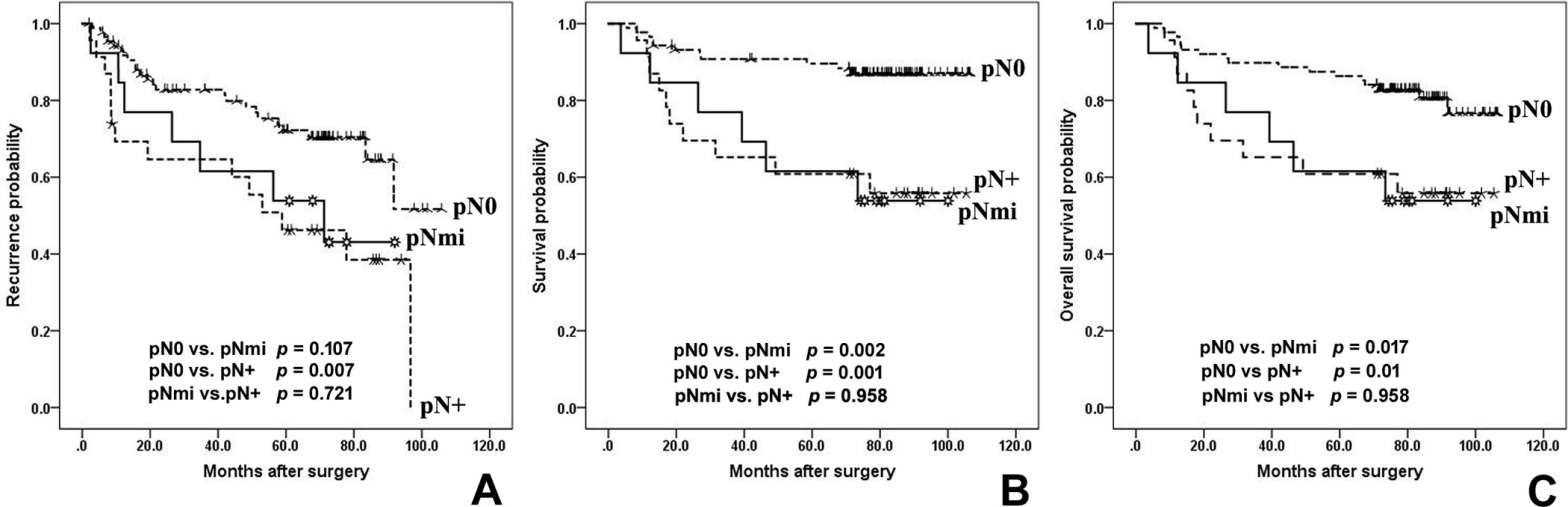
Recurrence-free survival (A), cancer-specific survival (B), and overall survival (C) based on pN stage. pN0: the cases without nodal metastasis after immunohistochemistry; pNmi: the cases with occult lymph node metastasis who were originally pN0 at the initial diagnosis; pN+: the cases with nodal metastasis in both initial and final diagnoses. Kaplan-Meier survival curves with log rank test are shown.

Five-year RFS rates of the pN0, pNmi, and pN+ groups were 89.6%, 61.5%, and 46.2%, respectively, with five-year CSS rates of 89.6%, 61.5%, and 60.9%, and five-year OS of 86.4%, 61.5%, 60.9%, respectively. Kaplan-Meier survival curve showed a significantly negative impact on CSS (*p* = 0.002) and OS (*p* = 0.017) in the pNmi group than in the pN0 group, and a worse trend in RFS with no significant difference (*p* = 0.107; Fig 3). The pN+ group also had significantly worse RFS (*p* = 0.007), CSS (*p* = 0.001), and OS (*p* = 0.01) than the pN0 group. However, clinical outcome was not significantly different between the pNmi and pN+ groups (RFS, *p* = 0.721; CSS, *p* = 0.958; OS, *p* = 0.958).

Since node metastasis affects the decision to perform AC at low tumor stage, the effect of occult LN metastasis on prognosis was analyzed in subgroups (pT1-2 and pT3-4 stage). No one received AC among pN0 and pNmi patients in the pT1-2 subgroup. It was found that 12.5% (11 of 88) in pN0 and 33% (3 of 9) in pNmi group died of the disease, whereas only 25% (1 of 4) of pN+ patients in the pT1-2 subgroup died of the disease. Among pNmi patients in the pT3-4 subgroup (n = 4), 3 patients received AC and 3 patients died of the disease. Among pN+ patients in the pT3-4 subgroup (n = 19), 9 patients died of the disease. All pN+ patients received AC in this study cohort. In both subgroups, there was no significant difference in RFS, CSS, or OS among pN0, pNmi, and pN+ groups (pT1-2 subgroup, *p* = 0.665, *p* = 0.076, and *p* = 0.457, respectively; pT3-4 subgroup, *p* = 0.137, *p* = 0.168, and *p* = 0.168, respectively).

Based on univariate Cox analysis, pN+ group (*p* = 0.008), higher pT stage (*p* = 0.042), advanced TNM stage (original version, *p* = 0.039; new version, *p* = 0.037), and necrosis (*p* = 0.004) were significantly associated with increased disease recurrence (Table 3). Regarding CSS, pNmi (*p* = 0.004) and pN+ (*p* = 0.001) groups, higher pT stage (*p* = 0.001), advanced TNM stage (original version, *p* = 0.001; new version, *p* < 0.001), mixed UC (*p* = 0.003), lymphovascular invasion (*p* = 0.003), budding-like clusters (*p* = 0.012), and necrosis (*p* = 0.001) were significantly associated with increased risk of event. In multivariate Cox analysis, pNmi group (CSS, *p* = 0.040), pN+ group (RFS, *p* = 0.013; CSS, *p* = 0.026; OS, *p* = 0.039) and higher T stage (RFS, *p* = 0.036; CSS, *p* = 0.002; OS, *p* = 0.006) independently increased the risk of disease recurrence and cancer mortality (Table 4). AC independently decreased the risk of disease recurrence (*p* = 0.028), cancer-specific death (*p* = 0.034) and overall death (*p* = 0.023). However, pNmi was not an independent prognostic factor for disease recurrence (RFS, *p* = 0.202) and overall death (*p* = 0.128).

**Table 3:** Univariate Cox analysis for recurrence-free survival, cancer-specific survival, and overall survival.

Clinicopathological parameters		Disease recurrence			Cancer-specific death			Overall death		
		HR	95% CI	p values	HR	95% CI	p values	HR	95% CI	p values
Age (years, continuous)		1.014	0.985–1.044	0.334	1.030	0.99–1.071	0.140	1.034	0.998–1.072	0.068
pN stage										
	pNmi vs. pN0	2.025	0.871–4.709	0.101	4.234	1.565–11.458	0.004	2.888	1.137–7.336	0.026
	pN+ vs. pN0	2.452	1.266–4.749	0.008	4.099	1.739–9.662	0.001	2.709	1.239–5.924	0.013
pT stage										
	≥pT3 vs. ≤pT2	1.842	1.022–3.32	0.042	4.002	1.83–8.75	0.001	2.591	1.307–5.137	0.006
Original pTNM stage										
	III-IV vs. I-II	1.851	1.031–3.326	0.039	4.099	1.84–9.133	0.001	2.519	1.268–5.004	0.008
New pTNM stage										
	III-IV vs. I-II	1.878	1.039–3.397	0.037	5.245	2.116–13.002	<0.001	2.701	1.328–5.493	0.006
Histologic subtype										
	Mixed vs. pure conventional	1.495	0.832–2.688	0.179	3.761	1.588–8.903	0.003	2.748	1.33–5.678	0.006
Tumor grade										
	High vs. Low grade	0.863	0.414–1.796	0.693	0.705	0.285–1.748	0.451	0.758	0.329–1.748	0.516
Lymphovascular invasion										
	Present vs. absent	1.722	0.957–3.096	0.070	3.303	1.482–7.361	0.003	2.613	1.298–5.26	0.007
Perineural invasion										
	Present vs. absent	1.311	0.583–2.946	0.513	2.144	0.864–5.32	0.100	2.086	0.904–4.814	0.085
Budding-like clusters										
	Present vs. absent	1.964	0.972–3.968	0.060	4.629	1.393–15.379	0.012	2.196	0.952–5.064	0.065
Necrosis										
	Present vs. absent	2.527	1.355–4.712	0.004	3.534	1.636–7.634	0.001	2.599	1.271–5.314	0.009
Adjuvant chemotherapy										
	Present vs. absent	1.327	0.725–2.428	0.359	2.087	0.981–4.441	0.056	1.492	0.742–3.001	0.262

**Table 4:** Multivariate Cox analysis for recurrence-free survival, cancer-specific survival, and overall survival.

Clinicopathological parameters		Disease recurrence			Cancer-specific death			Overall death		
		HR	95% CI	p values	HR	95% CI	p values	HR	95% CI	p values
pN stage										
	pNmi vs. pN0	1.792	0.732–4.388	0.202	2.97	1.050–8.404	0.040	2.143	0.802–5.725	0.218
	pN+ vs. pN0	5.006	1.408–17.804	0.013	4.601	1.198–17.669	0.026	4.008	1.074–14.963	0.039
pT stage										
	≥pT3 vs. ≤pT2	2.771	1.069–7.184	0.036	6.02	1.921–18.859	0.002	4.412	1.543–12.62	0.006
Histologic subtype										
	Mixed vs. pure conventional	1.223	0.631–2.371	0.550	2.205	0.862–5.638	0.099	1.874	0.839–4.187	0.126
Adjuvant chemotherapy										
	Present vs. absent	0.204	0.05–0.839	0.028	0.206	0.048–0.889	0.034	0.188	0.045–0.795	0.023

## Discussion

In several studies, IHC was used to detect LN micrometastasis in UBC patients undergoing RC. Yang *et al*. [5] primally found only one micrometastasis (0.62%) among 159 negative LNs after CAM5.2 and AE1AE3 IHC in high-grade muscle-invasive UC of the urinary bladder. This corresponded to 1 (5.6%) out of 19 patients who were originally N0 with routine H&E staining. The authors concluded that standard H&E staining would be adequate and that routine IHC was not useful for nodal staging in UBC, although survival analysis was not performed [5]. Jenson *et al*. reported that micrometastasis was found in 1 (0.56%) out of 173 negative LNs in pT1-T3 UBC (corresponded to 1 out of 10 patients) and that did not correlate with survival or prognosis [6]. In a prospective study, Matsumoto *et al*. found micrometastasis in 4 (8.5%) out of 47 pN0 patients who underwent RC with extended lymphadenectomy [7]. However, the 2-year RFS was not significantly different between node-negative and micrometastasis groups after IHC [7]. Recently, Cuck *et al*. found that 2 out of 61 patients (3.3%) showed micrometastasis in muscle-invasive UBC. However, clinical outcome could not be evaluated because the patients died due to postoperative problems [8]. Studies of micrometastasis detected by real-time reverse transcription-PCR using RNA extract from LN showed that PCR-positive cases (micrometastasis) were found in 20–35% in the originally node-negative group by standard pathological examination [11–14]. The CSS of this micrometastasis (pNmi) group was significantly lower than that of the pN0 group in the univariate analysis, although there was no statistical significance in the multivariate analysis [11–13]. Gazquez *et al*. have found RT-PCR-detected micrometastasis in 25.7% (19 of 74 patients) of node-negative group by conventional histological analysis. After 100 months of follow-up, they found a low trend for RFS and CSS. However, they showed no statistical significance [14].

Because previous studies showed incomplete results, the prognostic significance of occult LN metastasis was investigated in the present study with a larger study cohort of UBC patients (n = 124) with longer follow-up period (median, 80 months) than previous studies. Pan-cytokeratin IHC showed newly identified microscopic LN metastasis in 12.9% of the originally node-negative patients and in 23.1% of the originally node-positive patients. The pathologic node stage was upstaged in 11.4%. UBC patients with occult LN metastasis (pNmi) had significantly worse CSS than truly node-negative patients (pN0) and survival curves of pNmi were similar to node-positive patients (pN+). In the pT1-2 subgroup, more patients in pNmi than in pN+ (33% vs 25%) died of disease, although they showed no statistical significance probably due to small number of pNmi and pN+ cases in pT1-2 subgroup (n = 13). Since pNmi patients in the pT1-2 subgroup were initially reported as node-negative, they did not received AC, while all pN+ patients received AC. AC might be one of the reasons why clinical outcome was worse in pNmi than pN+ patients in the pT1-2 subgroup. Therefore, UBC patients (especially pT1-2) with occult LN metastasis might have to take adequate postoperative management similar to node-positive UBC patients. In this study, occult LN metastasis independently increased the risk of cancer mortality in UBC patients. Among all patients, the pNmi group showed worse clinical outcome than the pN0 group in RFS, CSS, and OS. However, only CSS was statistically significant. In addition, the pNmi group was not an independent risk factor for RFS, CSS, or OS. Because the pNmi group included many early stage cancer patients, a sufficient follow-up period is critical to reveal prognostic significance of occult LN metastasis. In case of breast cancer, studies with follow-up period over 20 years showed that axillary LN micrometastasis was correlated with survival [15, 16], while studies with follow-up period of 5–8 years failed to reveal prognostic relevance of axillary LN micrometastasis [17, 18].

Performing IHC in LNs of all UBC cases is not practical to detect occult LN metastasis because of a significant additional cost. Therefore, it is necessary to select cases that have histologic features related to occult metastasis. So far, the relationship of occult LN metastasis with histopathologic features of UBC had not been reported yet. In this study, mixed UC, UBC with discohesive pattern, glandular differentiation, and budding-like clusters were significantly associated with occult LN metastasis. If UBC has a mixed histology, discohesive pattern, glandular differentiation, or budding-like clusters, IHC might be helpful for determining occult LN metastasis.

High tumor budding is a poor prognostic factor in colorectal cancer. It is associated with lymph node metastasis [19]. There are a few studies on tumor budding in UBC. Measurement methods or criteria have not yet been established yet. Tumor budding was found in 87% of UBC when using cut-off of 5 or more clusters consisting of 5 or less cells in the 400-fold field of view [20]. It was 17.4% when using cut-off of 10 or more clusters consisting of 5 or less in the 200x field of view[21] and 73.6% when using cut-off of 14 budding in the 400x field of view [22]. These studies have reported an association of tumor budding with a low survival rate. In UBC, it is difficult to access tumor budding by the criteria used in colorectal cancer. First, the invasive front is usually unclear in UBC, for a tumor that is fragmented in a TUR specimen. In some cases, the residual tumor is absent in the RC specimen after TUR. Second, UBC histology often shows small tumor nests. Recently, poorly differentiated cluster (a tumor cluster defined as 5 or more tumor cells without gland formation) has been reported to be associated with a poor prognosis in colorectal cancer [23, 24]. Accordingly, we evaluated clinicopathological significance of poorly differentiated clusters in UBC. In this study, poorly differentiated clusters was named budding-like clusters and defined by isolated single tumor cells or small clusters composed of fewer than 20 cells among tumor area in more than one field of 200x magnification. Budding-like clusters was significantly associated with LN metastasis including occult LN metastasis in UBC.

In conclusion, cytokeratin IHC identified nodal micrometastasis and ITC in 12.9% of the originally node-negative UBC patients. Nodal micrometastasis and ITC independently increase the risk of cancer mortality in UBC. However, those with occult LN metastasis showed lower survival tendency in the pT1-2 subgroup. In the future, clinical significance of occult LN metastasis in early stage UBC should be clarified by larger scale studies. IHC might be selectively used to detect micrometastasis and ITC in UBC with specific pathological features such as mixed UC, UC with discohesive pattern, glandular differentiation, lymphovascular invasion, and budding-like clusters.
